# Supplementary material for: Laser nanofabrication inside silicon with spatial beam modulation and anisotropic seeding
Source: Nat Commun. 2024 Jul 16;15:5786. doi: 10.1038/s41467-024-49303-z (PMC11252398; doi:10.1038/s41467-024-49303-z)
Supplement: Supplementary file 1 — Supplementary Information [file 41467_2024_49303_MOESM1_ESM.pdf]

Laser nanofabrication inside silicon with spatial beam modulation and anisotropic seeding

SUPPLEMENTARY INFORMATION

Rana Asgari Sabet[1,2], Aqiq Ishraq[2], Alperen Saltik[1], Mehmet Bütün[1], Onur Tokel[1,2,†]

[1] Department of Physics, Bilkent University, Ankara, 06800, Turkey

[2] UNAM – National Nanotechnology Research Center and Institute of Materials Science and Nanotechnology, Bilkent University, Ankara, 06800, Turkey

[†]Corresponding author. E-mail: otokel@bilkent.edu.tr (O.T.)

Table of Contents

This supplementary information file contains Supplementary Notes 1-9.

Supplementary Note 1: Bessel beam creation with lens-virtual axicon doublets.

Supplementary Note 2: The role of seeding in the fabrication of volumetric nano-lines

Supplementary Note 3: Estimating the lithographic feature size (ξ) inside silicon

Supplementary Note 4: Vector correlations of scanning direction and laser polarisation in nanofabrication

Supplementary Note 5: Multi-level in-chip nanofabrication

Supplementary Note 6: Quantitative phase microscopy analysis

Supplementary Note 7: Two-layer sub-micron gratings for tuneable spectral response

Supplementary Note 8. Nanopatterning with sub-micron modulation

Supplementary Note 9. Depth control of nanostructures

Supplementary Note 1. Bessel beam creation with lens-virtual axicon doublets

The laser beam modulation type and specific focusing conditions are critical for energy deposition in subsurface lithography. We simulated two holographic profiles of type $\phi(r) = \exp(\pm i2\pi r/r_0)$, assuming the same optical setup used in our experiments (Methods). The negative sign in the phase corresponds to a converging axicon (Figs. S1a, S1b); whereas the positive sign corresponds to a diverging axicon (Figs. S1c, S1d). Here, in contrast to an arrangement based on a physical axicon and a conventional 4-*f* system[1], strong correlation between the length of the Bessel zone (z_B) and the central core size (d_B) is significantly reduced. This provides an additional degree-of-freedom, which proves critical for the rapid fabrication of high-aspect-ratio nano-planes/nano-lines inside Si. In particular, simply by dynamically changing r_0 with SLM, one can create Bessel beams of increased aspect ratio in Si, without significantly increasing the beam core size (Figs. S1c, S1d).

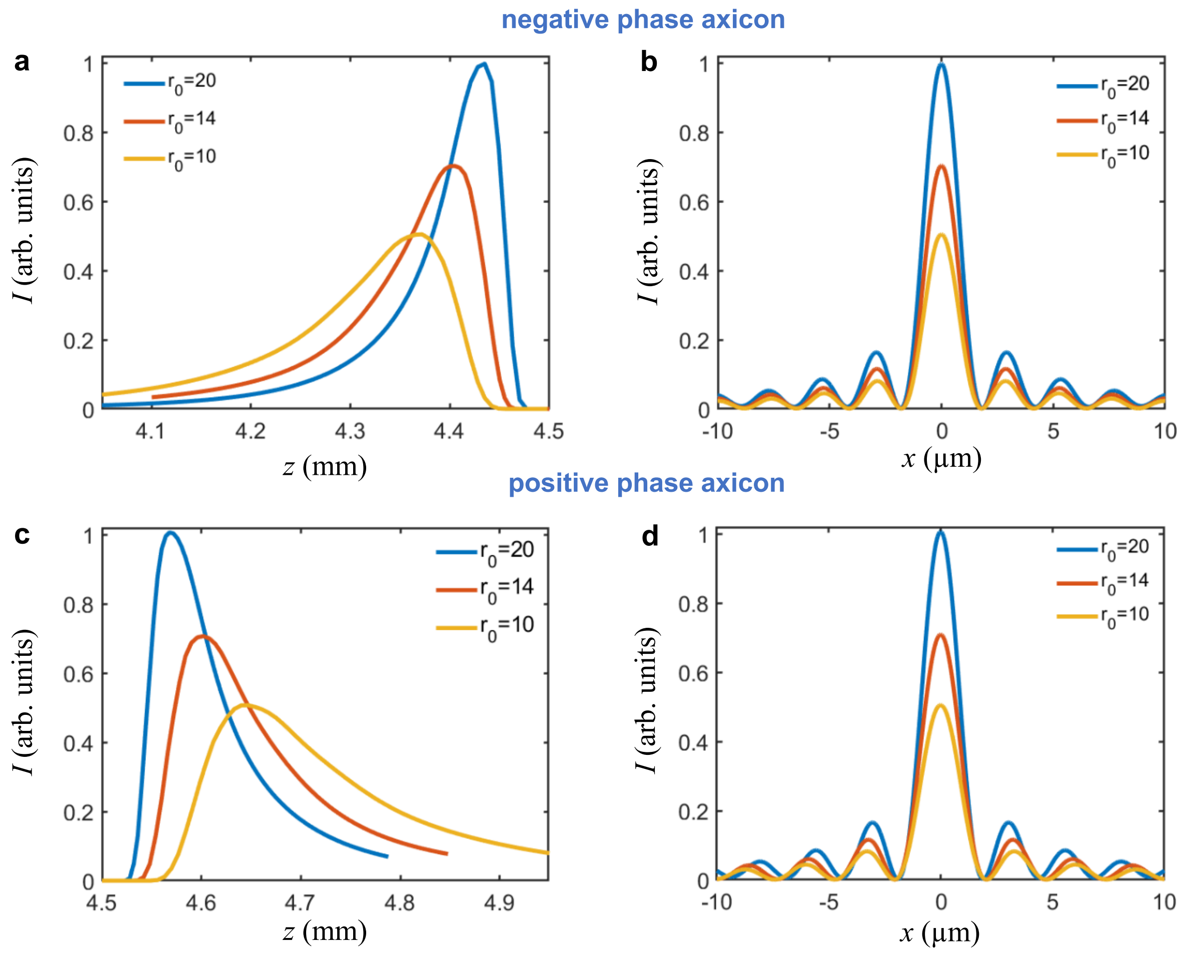

Figure S1: Simulated intensity patterns for positive- and negative-phase axicon - lens doublets for the same E_p. (a) The beam profile along the laser propagation axis for a negative phase axicon. **(b)** The transverse intensity profiles of (a) at the maximum intensity point. **(c)** The beam profile along the laser propagation axis for a positive phase axicon. **(d)** The transverse intensity profiles of (c) at the maximum intensity point. All distributions are normalised to the peak intensity of $r_0 = 20$.

In our experiments, as well as in the corresponding simulations of following sections, a positive-phase axicon-lens doublet is exploited (Figs. S1c, S1d), in order to achieve deeper subsurface lithography. This is because the selected arrangement presents lower intensity values before the focus. Such an energy deposition both prevents potential wafer surface alteration, as well as any subsurface alterations above the modifications; constituting a powerful feature of the method.

Supplementary Note 2. The role of seeding in the fabrication of volumetric nano-lines

We first show the creation of 2D-confined subsurface micro-structure arrays (*i.e.*, micro-lines) exploiting modulated laser beams. We use a Bessel beam propagating along the *z*-axis with $r_0 = 14$, $E_p = 10$ µJ, and irradiate the wafer for 30 s, creating micro-lines along *z* axis (Fig. S2a). Then, one may expect to simply reduce r_0 or E_p, in order to create volumetric nano-lines, however, such a programme does not reach the nanofabrication regime. For instance, $r_0 = 10$, $E_p = 10$ µJ allows for the creation of micro-planes or micro-lines, however reducing E_p, *e.g.*, $r_0 = 10$, $E_p = 4$ µJ allows for fabrication of nano-planes, but not for the creation of nano-lines (Similarly, reduced values for r_0 only allow for the creation of micro-lines).

Fortunately, there is a different lithography regime which can be exploited to fabricate nano-lines in Si. First, one creates a planar modification with a raster scan projection in the *x*-*y* plane (Fig. S2b, left). This layer, which may also be a nano-plane, is termed the preform or seed plane. Then, nano-line fabrication is initiated within a few µm region of this preform, simply by longitudinal scanning along the *z*-axis (Fig. S2b). Extensive observations indicate that low fluences or small r_0 values can create nano-lines near such preforms, whereas nano-lines do not form if one starts at an empty region using the same set of laser parameters. This is identified as far-field based or non-local seeding. Further, it is possible to control the anisotropy of nano-lines with the laser polarisation or extend them indefinitely with scanning (Figs. 3d-3e, Manuscript). This is identified as near-field based or self-seeding. This intriguing effect is discussed next.

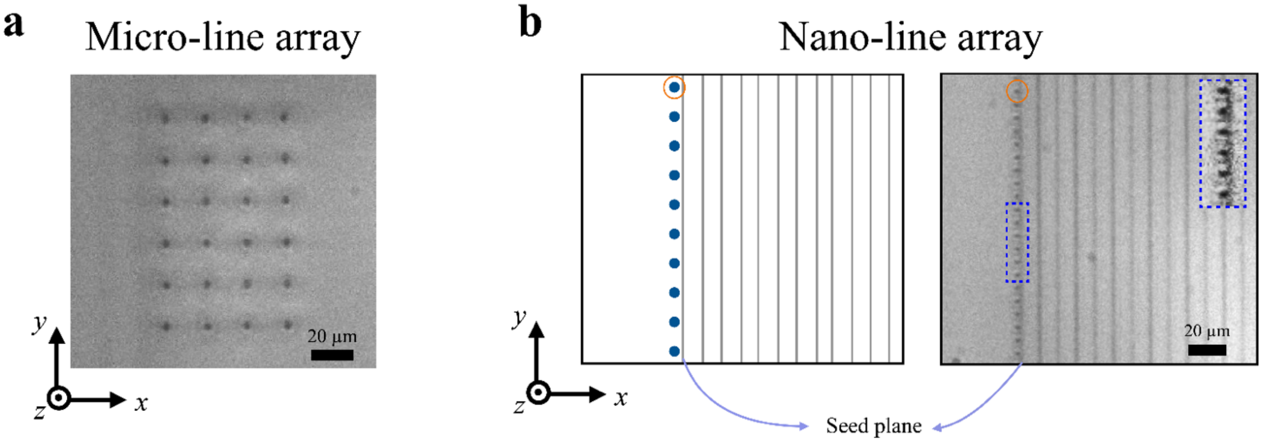

Figure S2: Laser lithography of micro- and nano-line arrays in Si. (a) Infrared transmission microscope (ITM) image of an array of micro-lines inside Si, created by irradiating with a Bessel beam of $r_0 = 14$, $E_p = 10$ µJ. **(b)** Schematic shows first, the creation of seed planes functioning as preforms, followed by creation

of an array of nano-lines in the proximity of the seed plane. ITM image shows fabricated nano-lines (orange circle), with the corresponding geometry given on the left. The seed micro-plane is fabricated with $r_0 = 10$, $E_p = 6$ µJ; whereas nano-lines are fabricated with $r_0 = 10$ and $E_p = 4$ µJ, and 2 µm away from the seed. The laser polarisation is along the y-axis in all experiments. The feature size are confirmed with SEM analysis. *Inset*: Contrast-enhanced and zoomed area illustrates the preform and six nano-lines together.

Discussion on near-field based feedback mechanisms and far-field regulation

Laser nanostructuring inside transparent materials generally requires the existence of seeds in the form of inhomogeneities, scattering centres, or voids[2]. The field, on or around such scattering centres is shown to be enhanced with characteristic near- and far-field patterns[3]. Indeed, analogous field enhancement effects are ubiquitous in laser-induced-periodic-surface-structuring (LIPSS)[3,4]; notably in nonlinear laser lithography of surfaces[5], where the laser polarisation establishes the symmetry of the entire nanopattern created over large areas[5]. A detailed understanding of responsible nonlinear feedback effects in the near- or far-field even allows laser nanopatterning of surfaces with all possible 2D Bravais lattice symmetries[6].

In the context of 3D laser lithography, *e.g.*, inside glasses, multi-photon absorption induces similar effects based on near- or far-field redistribution. These enable two distinct modes of highly-controlled laser-nano-fabrication, **(i)** asymmetric nanostructures originating from polarisation-dependent near-field scattering of nano-voids[7,8] **(ii)** far-field redistribution around previously formed structures, guiding the fabrication of neighbouring structures[7,9]. In 3D nanolithography of Si, we invoke analogous effects to explain the near-field based polarisation control of nano-lines (Fig. 3b, Fig. 3d of Manuscript); as well as the far-field based non-local seeding (Fig. 1b, Fig. 2d-i of Manuscript) observed in the emergence of nano-lines.

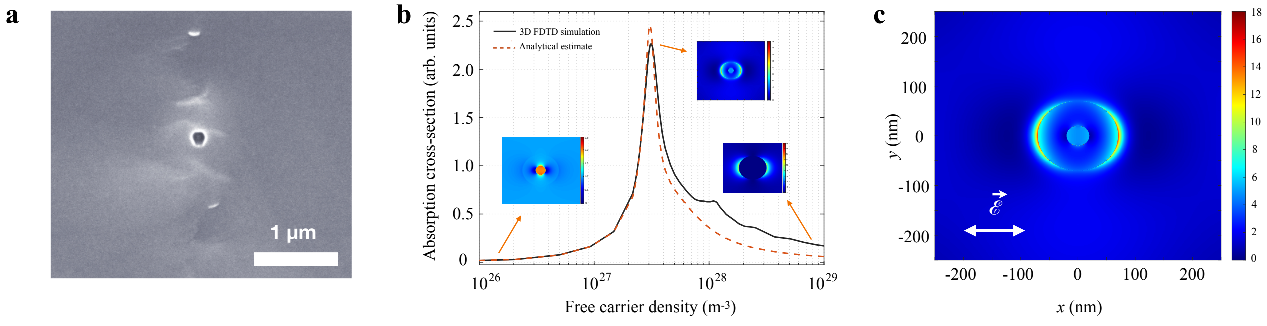

Figure S3: Localised field enhancement of a nanoscale void in Si. (a) Scanning Electron Microscope (SEM) image acquired from the cross-section of a subsurface modification, which involves a nanoscale void. **(b)** Three dimensional (3D) finite-difference-time-domain (FDTD) simulations for field redistribution

around a 40-nm diameter nano void in Si. The absorption peaks at the plasmon resonance frequency for $\rho = 3.07 \times 10^{27}$ m^{-3}. Inset: Intensity patterns at three different carrier densities. 3D FDTD simulations are performed with the quasistatic approximation, assuming Drude-Lorentz model with a collision frequency of 2×10^{13} s^{-1} and for a duration of 100 fs. The laser has a wavelength of $\lambda = 1550$ nm and is polarised along the x axis. The dotted curve is analytical estimate using Mie theory given for a spherical inhomogeneity. **(c)** The cross-section of intensity distribution calculated at the plasmon resonance. A charge density of $\rho = 3.07 \times 10^{27}$ m^{-3} is assumed to be distributed uniformly in a shell around the void, with a range comparable to the skin depth. The field enhancement is found to be parallel to the laser polarisation.

It is helpful to consider nano-line fabrication in two phases, *i.e.*, (i) "emergence" and (ii) "evolution", with distinct characteristics. This is a useful approach used in the study of nonlinear self-organised processes, which captures the salient features of the physics involved. Emergence is related to far field enhancement, as will be discussed later; whereas evolution is associated with inherent feedback mechanisms in the laser-material system. This behaviour is captured as "self-seeding" and is described next.

Near-field localisation and self-seeding: We presume laser-induced creation of nanoscale voids, *e.g.*, 20 - 100 nm voids at the early stages of fabrication. The existence of such structures are confirmed with SEM analysis, showing a distribution in laser-written parts (Fig. S3a). During irradiation, the free carrier density around a void strongly increases due to the positive feedback[10], between the field intensity and complex refractive index. This is due to the fact that the complex relative dielectric constant ($\varepsilon_r = \varepsilon_1 + i\varepsilon_2$) is linked to free carrier density. Assuming quasistatic approximation[10], we performed three-dimensional finite-difference-time-domain (FDTD) simulations for the field enhancement as a function of free carrier density, around a 40-nm spherical void in Si (Fig. S3b). The simulations are performed with Lumerical software, assume Drude-Lorentz model involving reduced mass for electron and holes, and collision time of 2×10^{13} s^{-1}. The absorption resonance is observed at the carrier density of $\rho = 3.07 \times 10^{27}$ m^{-3}. The corresponding intensity pattern indicates strong field enhancement parallel to the laser polarisation (Fig. S3c). Similar results are found for different void sizes, as well as non-void inhomogeneities with excess carrier densities.

We presume strong localised field enhancement results in further material modification, either by creating additional nano-voids, or even nanoscale inhomogeneities. Once the morphology of the scatterer is updated due to material modification, an updated field enhancement pattern would follow, which would again align parallel to laser polarisation. This would establish a further

positive feedback mechanism, *i.e.*, between the shape of the modification and the anisotropic field enhancement. This observation explains the observed morphology of the nano-lines, which are found parallel to laser polarisation (Fig. 3d, Manuscript). Finally, one can scan the laser away from such areas, where the near-field enhancement allows effective threshold reduction and elongation of nano-lines hundreds of micrometres away from the initial structure, as well as away from the preform (Fig. 2c-ii). We identify this complex set of processes simply as "self-seeding".

Far-field enhancement and emergence: We invoke far-field enhancement from the preform plane in order to explain the emergence of nano-lines (Fig. 1b). Analogous field redistribution effects around various preform structures have been successfully exploited to create 40-nm nano-lines buried inside silica glass[7], 20-nm feature sized lines on dielectric surfaces[11], and nanopatterns over silicon thin films[12]. Here, the preform which includes voids (or inhomogeneities) inside, helps enhance the field within a few micrometre range, effectively reducing the threshold for the creation of nano-lines. After the emergence of the nano-lines, laser-material system follows the near-field based feedback processes described before.

Scaling of nano-scale voids: Strong beam delocalization is associated with complex nonlinear interactions observed with intense femtosecond pulses inside the semiconductor[13-15], which has so far prevented robust 3D micro-structuring in Si[13]. This has previously been overcome by using nanosecond pulses of Gaussian profile, where nonlinear-feedback-mechanisms initiate and enforce the beam collapse[16]. However, structuring is still limited to micro-scale, both in single- and multi-beam experiments[13]. This is in part due to creating large scattering centres or voids during material modification. If nano-scale voids can be created, these may assist in field enhancement and scattering, as further discussed below.

Scaling of voids

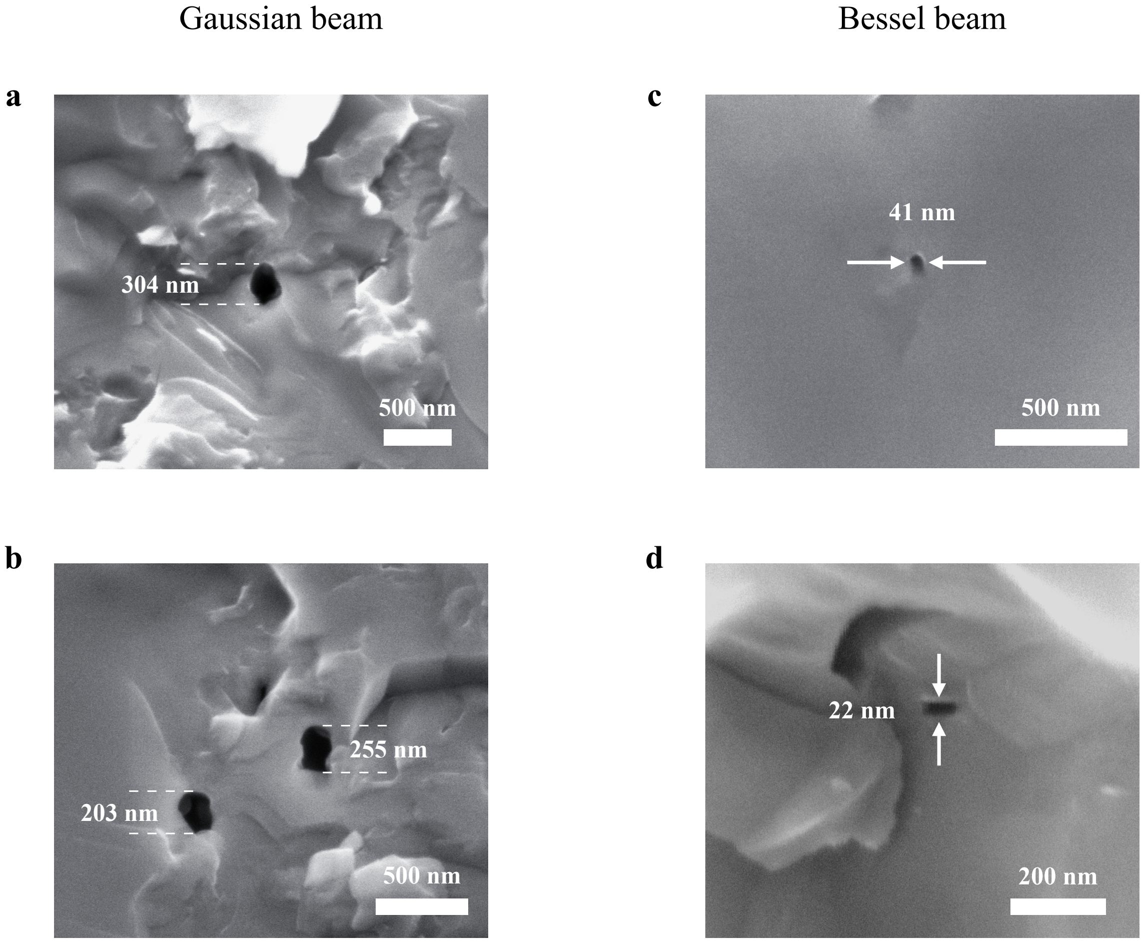

Figure S4. **The scaling of subsurface void size with the beam modulation**. **(a, b)** Scanning electron microscopy (SEM) images of subsurface voids within modified areas. These voids are created with Gaussian beams and can form at scales of hundreds-of-nanometres. **(c, d)** SEM images subsurface voids within modified areas. These are created with Bessel type beams and are observed to be of few tens-of-nanometres size. All experiments are performed with pulse energy of E_p = 5.6 µJ, scanning speed of 2 mm/s, and horizontal polarisation. Bessel beam is created with $r_0 = 6$. The SEM data is recorded directly from the wafer cross-sections after cutting; before any etching or post-processing steps on samples. Measured feature sizes are, ξ = 3 µm in (a,b), and ξ = 370 nm in (c,d).

We performed extensive laser-writing experiments using Gaussian and Bessel beams, in order to evaluate such subsurface voids (Fig. S4). Figs. S4a-S4b show representative voids observed inside modified areas, written with Gaussian beams of E_p = 5.6 µJ, scanning speed of 2 mm/s, horizontal polarisation, and single-beam writing. While the modified section is of ξ = 3 µm, the voids are observed to be ~ 300 nm (Fig. S4a), or consist of neighbouring voids of >200-nm size (Fig. S4b).

This observation is in stark contrast compared to the results of Bessel beam experiments (Figs. S4c-S4d). In these experiments, we used the same laser parameters except the beam modulation, which reduced the feature size to ξ = 370 nm. Further, the size of the voids were observed to be on the 20 – 40 nm scale (Figs. S4c-S4d). *We note that these are the smallest-sized voids so far observed buried inside Si crystals*. The transition from micro-structuring to nano-structuring is found to be self-consistent, in the sense that the void inside the modified area is not larger than the feature size, ξ.

Thus, while the robust energy localization during material modification is sustained due to the remarkable non-diffracting and self-healing characteristics of Bessel beams[17,18], and the nonlinear self-focusing is achieved due to thermal lensing[16], one may also expect strong field enhancement due to the emergence of nano-scale voids. These also reduce the beam scattering in comparison to the larger-sized voids found in Gaussian-beam experiments (beam scattering scales with the sixth power of the scatterer size in Rayleigh scattering). The nano-voids continually modify and regulate the complex evolution of the system, based on field enhancement. While the complex nonlinear dynamics of feedback-based interaction is beyond the scope of this work, in order to gain further insight, we performed detailed simulations for the field enhancement, which varies as a function of the nano-void size. The associated simulations are further discussed below.

Field enhancement of nano-voids: The exact nature of the emergence of nano-voids is currently unknown. However, as they form during material modification, they create strong field enhancement in their immediate environment, analogous to plasmonic nano-particles. We confirm this behaviour with 3D finite-difference-time-domain (FDTD) simulations using Lumerical™ solver (Fig. S5). We assume nano-voids of varying sizes (2 nm to 300 nm), with a uniform carrier density of $\rho = 3.1 \times 10^{27}$ m^{-3} of the same size surrounding voids, corresponding to the peak of absorption in Fig. 1c of the Manuscript. The field enhancement ($|E|^2/|E_0|^2$) is evaluated along a linear cross-section, parallel to the beam polarisation axis. The field is found to be significantly enhanced for small scattering centres, in particular for the sub–40 nm sized voids (Fig. S5). This size range matches to the observations of voids in the Bessel beam experiments (Figs. S4c-S4d). Further, the enhancement is almost entirely absent for larger sized voids, over a few hundred nanometre range (Fig. S5). This strongly suggests that as nano-voids emerge at the 20 – 40 nm scale, they would be regulated in size. In contrast, there would be no such regulatory mechanism

for the > 250-nm sized voids, in particular for those experimentally observed within the modifications of Gaussian beam experiments (Figs. S4a-S4b).

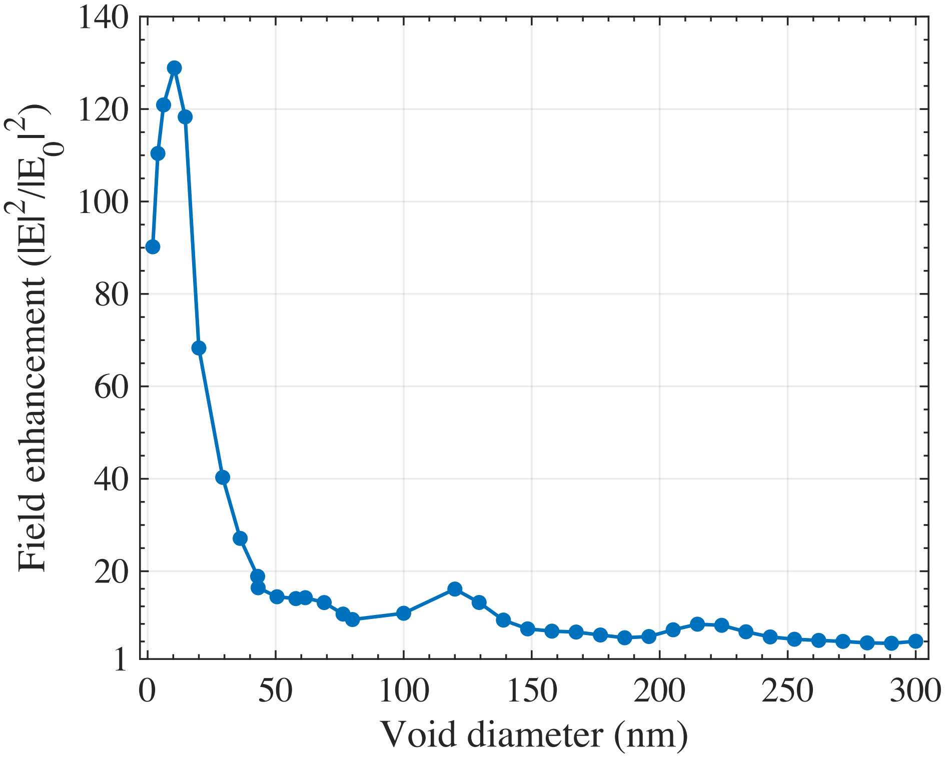

Figure S5. Local field enhancement as a function of the void diameter. 3D finite-difference-time-domain (FDTD) simulations are performed for evaluating the field distribution around spherical voids. The simulations are performed with Lumerical™ solver, using the Drude model. The void diameter is scanned from 2 nm to 300 nm, for 40 data points. We assume a uniform carrier density of $\rho = 3.1 \times 10^{27}$ m^{-3}, corresponding to the peak of absorption in Fig. 1c of the Manuscript. The size of this region is the same as the void size. The simulations are performed on an Intel processor (i7-10700F CPU 2.90GHz), over a time period of 180 hours. The mesh size is chosen as 0.2 nm – 5 nm.

Thus, significant field enhancement is expected in Bessel beam experiments, which is considered to effectively reduce the modification threshold during laser writing. Further, the field enhancement is anisotropic as illustrated in Figure 1 of the Manuscript, which also explains the polarisation dependency of the nano-lines morphology observed in our experiments (Figure 3, Manuscript).

Supplementary Note 3. Estimating the lithographic feature size (ξ) inside silicon

The interaction of laser with Si within the bulk of the wafer has been shown to be highly nonlinear, involving temporal feedback mechanisms and complex formation dynamics[16]. Here, we do not attempt to model the detailed evolution of nanostructures, but use a simple thresholding-based nonlinear model to guide our experiments. In the model, we define the feature size (ξ) as the length of the section that remains above a given modification intensity threshold (I_{th}) (Fig. S6a).

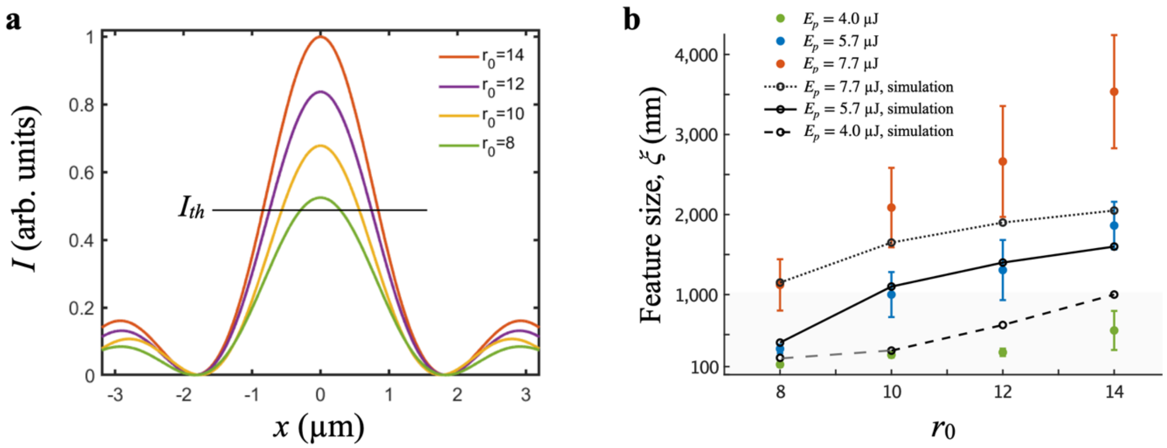

Figure S6: Feature size as a function of r_0 and pulse energy E_p. (a) Transverse beam profile for Bessel beams of r_0 = 8, 10, 12 and 14, focused into the middle of wafer (d_{nom} = 90 μm). The pulses have the same energy, with peak intensity for r_0 = 14 normalised to unity. We define the part above threshold (I_{th}) as the feature size (ξ). **(b)** Experimental feature sizes compared to corresponding simulations, given as a function of E_p and r_0 for d_{nom} = 90 μm (E_p = 4 μJ, r_0 = 8 assume d_{nom} = 170 μm). The filled circles of green, blue and orange colour are reproduced from Fig. 3a of the Manuscript, and are compared to model predictions given with black circles. Threshold, I_{th}, is treated as a free parameter and estimated from E_p = 5.7 μJ data. Transverse profiles for other E_p are found by integrating and scaling with respect to corresponding r_0 values.

Since lower r_0 values would correlate with deeper laser lithography (Fig. S1c), we also include spherical aberration term in the model (Fig. S6a) by appending the phase term[19,20],

$$\emptyset_{SA}(r) = \exp\left(i \frac{2\pi d_{nom}}{\lambda_0 f}\left[\sqrt{f^2 n_2^2 - r^2} - \sqrt{f^2 n_1^2 - r^2}\right]\right),$$

where f is the focal length of the lens, n_2 is the refractive index of Si, n_1 is the refractive index of air, and r is the radial coordinate, λ_0 is wavelength in air, and d_{nom} is the nominal focus. The transverse profiles at axial maximum intensity for d_{nom} = 90 μm are given in Fig. S6a. It is seen

that smaller r_0 is associated with modest narrowing in core size (Fig. S6a), in comparison to that of Fig. S1d. Then we apply the model to estimate feature sizes (ξ) with experimental parameters (Fig. S6b) and compare these with ξ recorded as a function of E_p and r_0 (Fig. 3a, Manuscript).

The model compares well with the experiments, in particular for the nanofabrication regime (Fig. S6b). In contrast, for the micro-fabrication regime, *i.e.*, E_p = 7.7 μJ, deviation from the predictions increases for higher r_0 values. We ascribe this difference to intensity values well-above the I_{th}; introducing micro-explosions enlarging the modification size. On the other extreme, one finds experiments with lower E_p and r_0. For instance, E_p = 4 μJ, r_0 = 8 requires deeper focusing in order to reach the I_{th} value. This is achieved with some support from spherical aberration which increases the peak intensity, resulting in highly reproducible subdiffraction nanostructures (Fig. S6b).

We also compare the prediction of ξ as a function of E_p, for r_0 = 10, with the experimental results given in Figure 4a, Manuscript (Fig. S7). While the model ignores the effect of laser polarisation, it compares well with our experiments, providing a strong guide for laser nanolithography.

These observations also suggest that r_0 and E_p can be used as effective control parameters for subsurface nanolithography of Si.

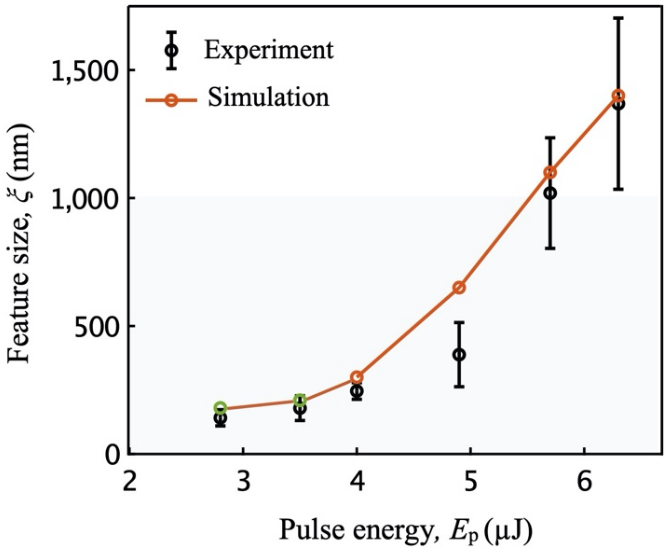

Figure S7: Feature size (ξ) as a function of pulse energy (E_p). Experimental data from Fig. 4a, Manuscript (black circles) is compared with the model predictions (orange circles, d_{nom}= 90 μm; green circles, d_{nom}= 140 - 165 μm indicating deeper lithography). Error bars indicate standard deviation.

Supplementary Note 4. Vector correlations of scanning direction and laser polarisation in nanofabrication

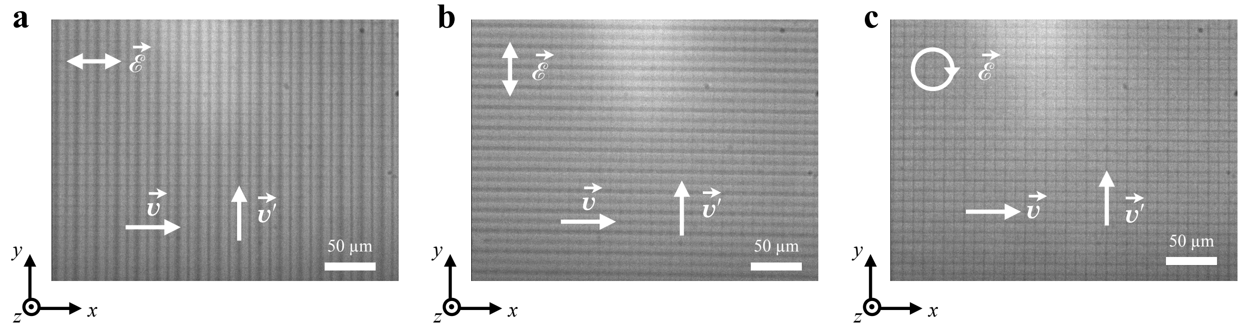

Figure S8: Vector correlations of scanning direction and laser polarisation in nanofabrication. Infrared transmission microscope images of 1D-confined subsurface structures, *i.e.,* subsurface planes, created with **(a)** horizontal, **(b)** vertical, and **(c)** circular laser polarisations. In all cases, the laser propagates along the z-axis, and the lithography is exploiting the parameters of $r_0 = 7$, $E_p = 8.5$ µJ. In both (a) and (b), when $\vec{E} \,//\, \vec{v}$, one observes reduces feature sizes (~ 500 nm), compared to the case of $\vec{E} \perp \vec{v}$ (~ 1500 nm). In comparison, when the laser polarisation is circular, the pattern is symmetric with a feature size corresponding to the mean of the preceding cases (~ 1000 nm). Scanning direction for individual subsurface planes are given with white arrows.

Supplementary Note 5. Multi-level in-chip nanofabrication

An exciting prospect is multi-layered laser-nanopatterning capability inside the wafer. In order to increase the length of modifications and thus, to increase their aspect ratio, we first create one level (Layer 1), followed by the second one (Layer 2), through coherent stitching of each layer to the next one (Fig. S9). Such structures are later used as volume Bragg gratings (Fig. 5, Manuscript and Suppl. Note 7). The SEM images showing single- and two-layer nanopatterns in Si are given in Figs. S9a-S9b. The periodicity of individual nano-planes is $\Lambda = 1.5$ µm, the average feature size is $\xi = 700$ nm. A magnified SEM image is shown in Fig. S9b, inset.

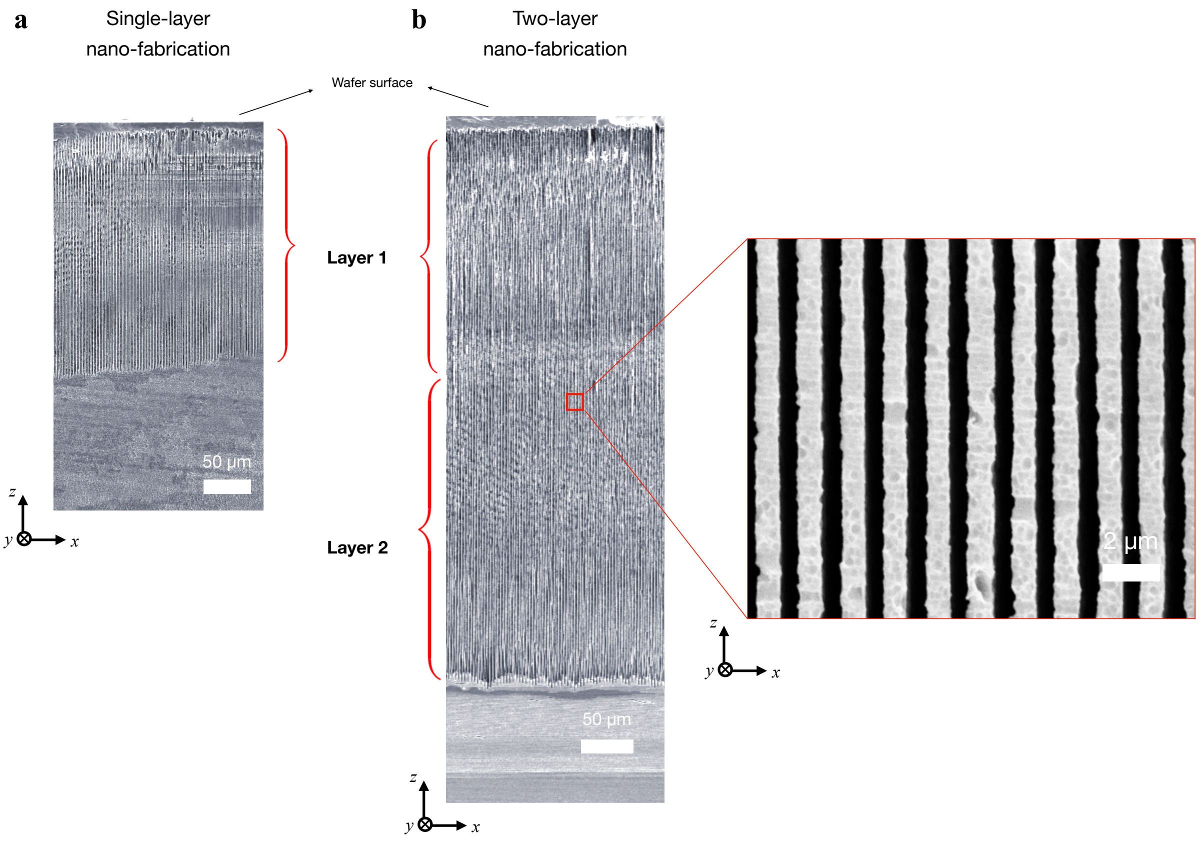

Figure S9: **Large-volume multi-level nanofabrication inside Si. (a)** Wide field-of-view SEM image of a single-layer nanopattern in Si. **(b)** Two-level coherently stitched nanopattern, created with $\xi = 700$ nm and $\Lambda = 1.5$ µm. The structures are fabricated using $r_0 = 7$ and $E_p = 8.7$ µJ. The laser propagates along the z-axis. The laser polarisation and scanning direction are both along the y-axis. *Inset*: Magnified view of a representative section in (b), after brief chemical etching in order to reveal the pattern.

Supplementary Note 6. Quantitative phase microscopy analysis

***In-situ* optical index analysis of lithographic structures.** It is possible to exploit quantitative phase microscopy (QPM) to directly measure the optical index of subsurface micro-patterns in Si[21,22]. We use a home-built QPM setup operating in the off-axis modality, with a lateral resolution of 1.5 µm and depth-of-field (DOF) of 6.5 µm. The schematic of the phase microscope is shown in Fig. S10a.

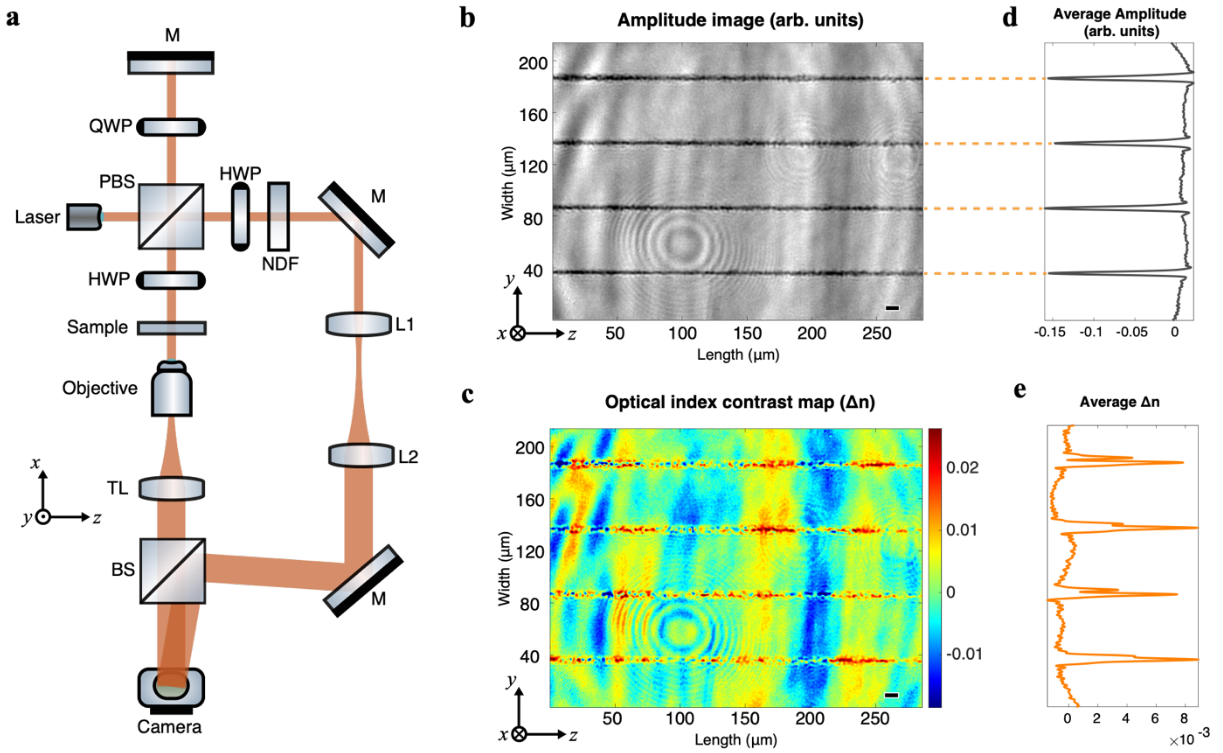

Figure S10: *In-situ* refractive index characterisation of subsurface lithographic patterns. (a) Schematic of the quantitative phase microscope system. M: mirror, QWP: quarter-wave plate. PBS: polarising beam splitter. HWP: half-wave plate. TL: tube lens. BS: beam splitter. L1, L2: lenses. NDF: neutral density filter. **(b)** Amplitude microscope image of buried structures acquired without interferometry. **(c)** Refractive index contrast map (Δn) acquired with QPM in off-axis mode. **(d)** The mean value for each row of the image given in (b). **(e)** The mean optical index contrast for each row of image given in (c). Scale bars: 10 µm.

Laser-written structures buried in Si are required to remain within the lateral resolution of the microscope, and should not extend along the propagation axis (x-axis) of the imaging laser more than the DOF. This is achieved by fabricating 2.5 ± 0.5 µm diameter lines (Fig. S10b), exploiting longitudinal writing. We used diced and polished Si samples extending 3-mm along z-axis. The fabrication laser is of E_p = 9 µJ, r_0 = 20, propagates along z-axis and is of linear polarisation (y-axis). The fabricated structures of rod-like symmetry cross the entire sample.

The patterned Si sample is then imaged with the QPM using off-axis interferometry, using a 20× magnification, NA = 0.4 objective (LMH-20X-532, Thorlabs). The imaging laser (1058-nm custom-built continuous wave laser) propagates along the x-axis to cross Si sample (Fig. S10a), and is polarised along the y-axis before the sample. The total field is recovered from the interference image, the phase is flattened by subtracting a reference measurement and polynomial surface fit.

The resulting amplitude and optical index contrast maps are shown in Fig. S10b and Fig. S10c, respectively. The corresponding averaged plots obtained along the length of modifications are given in Fig. S10d and Fig. S10e. We observe that optical index contrast (Δn) is fairly regular. We evaluate a Δn for these laser-written micro-structures as (7.3 ± 1.5) × 10^{-3}, noting that the value is a parameter of laser polarisation and energy delivery.

Birefringence & stress analysis. The phase microscope (Fig. S10a) can also be used to reveal the birefringence of modifications with associated stress patterns. First, two orthogonal-polarisation images (y and z axes) are acquired by rotating the half-wave plates in the arms of the interferometer. Then, by evaluating the difference between phase images, one can create a map of induced birefringence. Further, birefringence may be related to stress using Photoelastic Law[23-25].

$$\Delta\sigma = \sigma_{11} - \sigma_{22} = \frac{2(n_1 - n_2)}{{n_0}^3 \pi_{44}} = \frac{\Delta\varphi}{\pi d {n_0}^3 \pi_{44}}$$

where, $\Delta\sigma$ is the stress difference, $\Delta\varphi$ is the phase difference between two orthogonal polarisations, π_{44} is stress optical coefficient, σ_{11}, σ_{22} are the first and second principal axis stresses, and n_1, n_2 are the first and second principal axis optical indices, respectively, n_0 is index of unmodified Si, and d is the modification length along the imaging axis (x axis). We assume no shear stress. Using this relation, we create stress maps around the structures elongating along the z axis with diameter 2.5 ± 0.5 µm, which are created using longitudinal writing modality, and r_0 = 20 and E_p = 9 µJ.

The laser-writing polarisation along y axis case is summarised in Fig. S11. The amplitude, birefringence ($\Delta\Delta n$) and stress magnitude ($|\Delta\sigma|$) maps are given in Fig. S11a, Fig. S11b and Fig. S11c, respectively. We compare the amplitude for an individual micro-line with associated stress pattern in Fig. S11d, indicating that on average width of stress is the same as the width of modification.

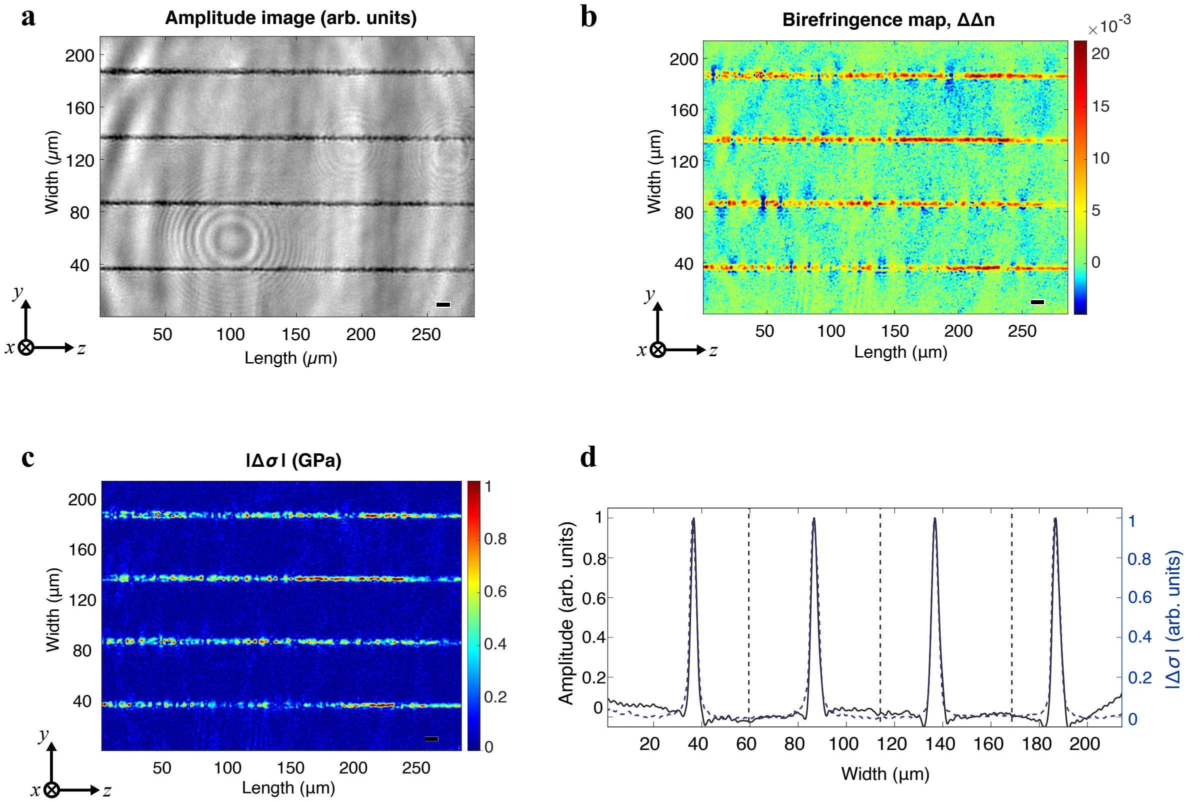

Figure S11: Stress map acquired with QPM for structures created with *y*-polarised laser. (a) Amplitude image recorded with IR microscopy. **(b)** The birefringence map. **(c)** The magnitude of stress map created using Photoelastic Law. **(d)** The mean value for each row in (c), compared to that of amplitude image given in (a). The stress (blue) and amplitude (black) curves are normalised for individual lines. Scale bars: 10 μm.

One may consider that potential residual stress around modifications may act as seed required to fabricate the nano-lines. In order to check this, we performed extensive experiments with up to 250 MPa stress in unmodified crystal Si, without observing any nano-lines in these regions. This strongly indicates that stress is not playing a significant role in the seeding effect. Nevertheless, observation of birefringence over these modifications, as well as potential residual stress around larger modifications, has implications for fabricating novel polarisation optics inside Si, *e.g.*, half-wave plates for near-IR[13]. In addition, the existence of stress and its gradient suggest the potential for modulation of higher-order optical nonlinearities, with novel applications[26-28].

A further interesting observation is that $\Delta n > 0$ for writing polarisation // imaging polarisation; whereas $\Delta n < 0$ for writing polarisation $\perp$ imaging polarisation. The covered range of Δn for modified parts was from -9×10^{-3} to 9×10^{-3} depending on fabrication laser polarisation. This wide index tunability has implications for in-chip refractive index engineering[13,29].

Supplementary Note 7. Two-layer sub-micron gratings for tuneable spectral response

We fabricated volume Bragg gratings (VBGs) with various feature sizes and periods, enabling different bandwidths. We use the multi-layer fabrication capability (Fig. S9) in order to fabricate large-volume-covering VBGs with ξ = 700 nm, Λ = 1.5 µm. The two-layer VBG (l = 490 µm) provided the highest diffraction efficiency (Fig. 5b, Manuscript). The corresponding spectral bandwidth is estimated as $\Delta\lambda$ = 27 nm. Through further decreasing feature size (ξ) and periodicity (Λ), one can achieve narrower spectral response. For instance, for VBGs fabricated with the geometrical parameters; ξ = 550 nm, Λ = 1.2 µm and l = 480 µm, the theoretical and measured spectral response ($\Delta\lambda$ = 14 nm) are found to be in strong agreement (Fig. S12). Still narrower spectral response is achieved exploiting gratings with ξ = 350 nm, Λ = 800 nm ($\Delta\lambda$ = 8 nm; Fig. 5d, Manuscript), indicating tuneable spectral response in Si.

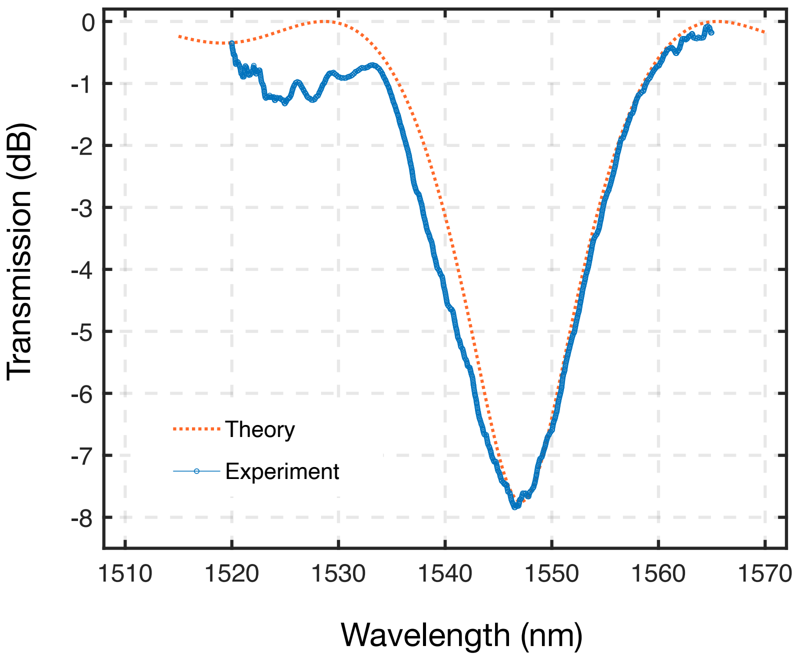

Figure S12: Spectral sensitivity of the VBG, created with ξ = 550 nm, Λ = 1.2 µm, and l = 480 µm. Bandwidth at λ_c = 1547 nm is measured as $\Delta\lambda$ = 14 nm. The VGB was fabricated with r_0 = 7 and E_p = 8 µJ. The diffraction efficiency is measured as %80 with a p-polarised λ =1.55 µm diode laser (Thorlabs, FPL1009S), and spectral response is measured with an amplified spontaneous emission (ASE) source[30].

Supplementary Note 8. Nanopatterning with sub-micron modulation

We conducted a systematic analysis, where nano-planes are fabricated with sub-micron periodicity. The nano-planes are fabricated with a Bessel beam of $r_0 = 7$, $E_p = 5.6 \pm 0.3$ µJ, $v = 1$ mm/s, and laser polarisation parallel to the sample scanning direction. The translation stage is set to create nano-planes with periodicities (Λ) ranging from 300 nm to 900 nm, followed by sample preparation for SEM analysis (see Methods). The SEM images are then analysed for periodicity and modulation error and the results are summarized in Figure S13. Here, each blue data point corresponds to the mean value that is calculated from a set of 100 measurements; and the associated error bar corresponds to the standard deviation from the same data set. The orange data points show experimental modulation error, which is calculated as the ratio of the standard deviation to the measured mean periodicity.

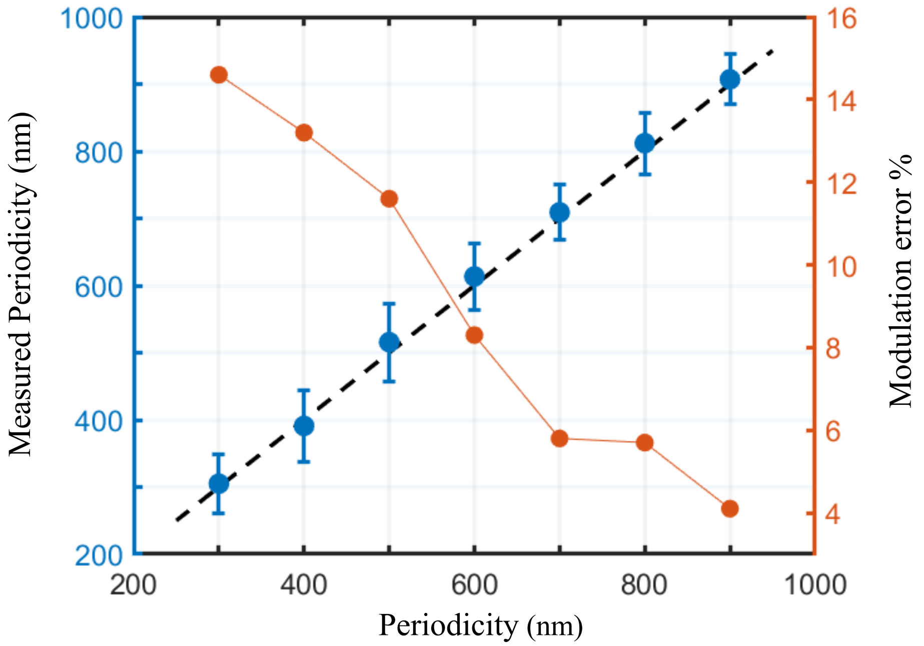

Figure S13: **Experimentally measured periodicity and associated modulation error**. Nano-planes are fabricated with different periodicities (Λ), using a Bessel beam of $r_0 = 7$, $E_p = 5.6 \pm 0.3$ µJ, $v = 1$ mm/s, and laser polarisation parallel to the scanning direction. The periodicity is varied from $\Lambda = 300$ nm to $\Lambda = 900$ nm, while keeping all other parameters the same. The blue data points correspond to the mean value calculated from a set of 100 data points which are extracted from scanning electron microscopy (SEM) analysis, whereas the error bars correspond to the standard deviation calculated for each data set. The orange data points indicate the error in periodicity (modulation error), which are calculated as the ratio of the standard deviation to the measured mean periodicity.

Figure S13 shows that the measured mean value of periodicity closely aligns with the target value. The dashed line, representing a curve of unity slope, visually illustrates this alignment. However, a decrease in periodicity is also accompanied with an increase in the modulation error (Figure S13). We further discuss roughness and reproducibly of these structures below. We first evaluate the measured roughness, followed by assessing the reproducibility at lowest values, *i.e.*, Λ = 300 nm.

Roughness analysis: Roughness is a useful metric in nano-fabrication. We use Average Roughness (R_a) definition, which is evaluated with the formula[31]:

$$R_a = \frac{1}{L}\int_0^L \left| X(z) - X_{\text{avg}} \right| dz \, ,$$

where X_{avg} is the mean of variable X calculated over a given range, $X(z)$ is the value of the variable for any given z position, and L is the length of the range under evaluation. R_a corresponds to the average deviation from the mean line. The implementation of this equation is illustrated schematically in Figure S14a, where the green curves correspond to the borders of modification, the red curve is the mean value of green curves at each z coordinate, and the orange line denotes the cumulative mean of all x coordinates (X_{avg}). Then, R_a may simply be found as the average of all deviations from this line (the blue line segments). We followed this methodology to calculate R_a values from SEM images of laser-written subsurface nano-planes. A MATLAB routine is written in order to achieve the required edge detection. A representative SEM image used for this approach is shown in Figure S14b, where the green and red curves have the same meaning as in Figure S14a.

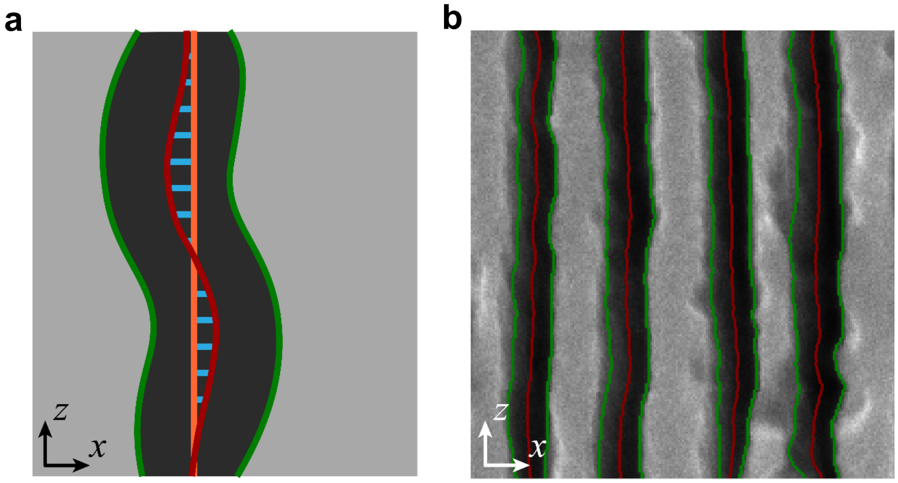

Figure S14: Definition and methodology for roughness, R_a. (a) Schematic for roughness calculation. Green curves follow the borders of modified parts, whereas their mean values for each z coordinate form the red curve. The orange line denotes the cumulative mean of all x coordinates (X_{avg}) of the red curve. The blue

horizontal lines show deviations from X_{avg} at each coordinate, ($|X(z) - X_{avg}|$). These are then used in the roughness calculation. **(b)** A representative SEM image of nano-planes, showing MATLAB edge detection (green curves) and associated mean value for each z (red curve).

Next, we performed a systematic analysis of R_a on nano-planes which are created with periodicities of Λ = 900 nm, 500 nm, 400 nm, and 300 nm. These structures are fabricated with Bessel beams in transverse writing modality, using $r_0 = 7$, $E_p = 5.6 \pm 0.3$ µJ, $v = 1$ mm/s and linear polarisation. The SEM images after the MATLAB colour coding for edge detection and mean analysis are shown in Figure S15a to S15d, with progressively reducing Λ to 300 nm. We observe that nano-planes in the Λ = 900 nm – 400 nm range form with high-quality; Λ = 300 nm planes are of partly reduced quality.

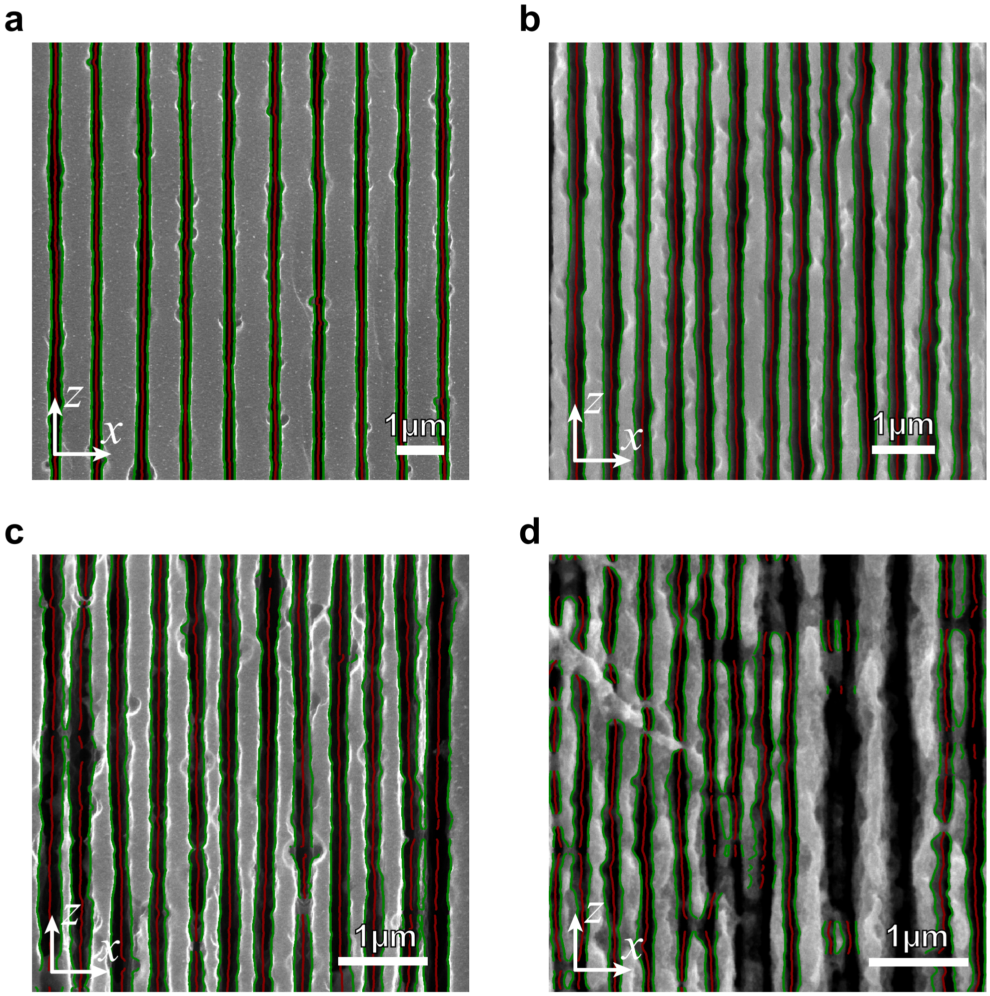

Figure S15: Roughness analysis. The SEM images are colour-coded with the same methodology described in Figure S14. **(a)** Λ = 900 nm, **(b)** Λ = 500 nm, **(c)** Λ = 400 nm, **(d)** Λ = 300 nm. The laser writing is performed with Bessel beams in transverse modality, using $r_0 = 7$, $E_p = 5.6 \pm 0.3$ µJ, $v = 1$ mm/s, and linear polarisation.

Using the SEM images in Figure S15, we evaluate the ξ and R_a parameters from > 7500 data points each, as summarized in the Table below.

Supplementary Table 1. Roughness analysis.

Periodicity, Λ (nm)	900	500	400	300
Feature size, ξ (nm)	210 ± 30	229 ± 34	187 ± 41	145 ± 36
Roughness, R_a (nm)	12	14.3	13.2	14.6

First, we observe that as Λ is reduced the expected periodicity is not affected (Figure S13); however the deviation in ξ tends to slightly increase (Supplementary Table 1). Second, the R_a value tends to remain within the 12–15 nm range, even down to Λ = 300 nm (Supplementary Table 1). Third, in the lowest modulation case of Λ = 300 nm (Figure S15d), some laser-written areas tend to merge, and nanofabrication uniformity and reproducibility may be reduced. While the non-diffracting nature of Bessel beams and their self-healing property sustain fabrication, such that the nano-planes form with expected periodicities (Figure S13), the merging indicates to a challenge in fabricating high-density nano-planes. This is attributed to beam distortion at very small modulation values, at scales when Λ becomes comparable to the expected feature size, ξ.

Roughness analysis over orthogonal cross-sections: Currently, it is not possible to cut a laser-modified volumetric nano-plane over its own plane at nanoscale, without introducing additional roughness. Thus, it would not be practical to directly evaluate R_a over a laser-written nano-surface. In order to gain further insight into the waviness of a buried nano-plane, a useful proxy is to evaluate R_a over two orthogonal cross-sections. We performed R_a analysis over two such perpendicular planes (x - z plane and x - y plane), which include the laser propagation direction (z direction) and the sample scanning direction (y direction), respectively. We fabricated subsurface nano-planes using r_0 = 6, E_p = 6.6 ± 0.3 μJ, Λ = 800 nm, v = 1 mm/s using linear polarisation parallel to the scanning direction. We then polished the sample (~ 150 μm) to reach the x - y cross-section of the nano-structures, followed by an orthogonal cut to reach their x - z cross-section. Brief etching is applied to reveal the nano-structures. For the nano-structures over the x - z plane, we evaluated the feature size as ξ = 142 ± 33 nm and the roughness as R_a = 19 nm. For the nano-structures over the x - y plane, we found ξ = 168 ± 31 nm,

with a roughness of $R_a = 18$ nm. Thus, similar feature size, standard deviation and roughness values are found for the nano-planes over two orthogonal cross-sections.

Straightness of translation stage: The straightness is defined as the displacement error perpendicular to the principal movement axis. For our stage (Aerotech, ANT130XY, ANT95LZ), the standard deviation of the displacement error is found using the stage feedback analysis tool as ~ 8 nm for 1-mm travel range, consistent with the factory datasheet. This value corresponds to less than 1 nm deviation for the preceding cross-sections analyses, too low to contribute to as error in R_a.

Supplementary Note 9. Depth control of nanostructures

One of the important requirements for 2.5D or 3D nanofabrication is depth control. We achieve this by positioning the onset of modifications to the desired depths along the laser propagation direction (z axis). This can be performed by varying the focusing depth in Si. In order to demonstrate this, we created an array of subsurface nano-planes in groups of ten, each group located at a progressively deeper position (Figure S16). In the experiment, the beam propagates along z-axis, where a $\Delta z = +5$ µm step in air corresponds to a depth change of $n_{Si} \times \Delta z$ (~ 17.5 µm) inside the wafer. This is confirmed by the measured change in the onset of nanostructures.

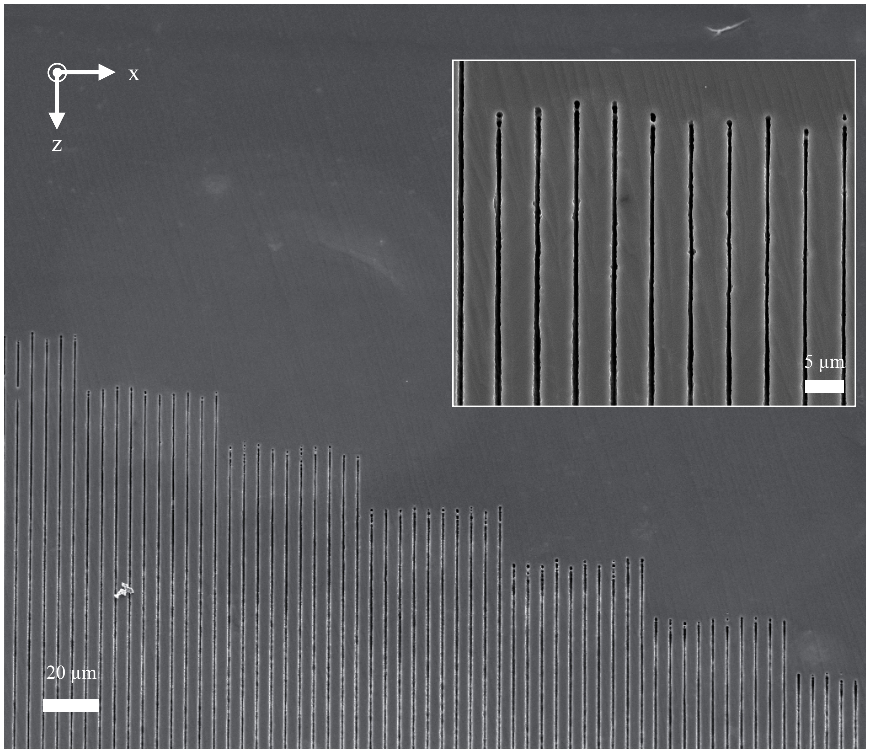

Figure S16: Nano-planes fabricated at different depths. Cross-sectional SEM image from a group of nano-planes, which are created at progressively deeper locations within the wafer. Each group of 10 nano-planes are written with 5-µm steps in air, corresponding $n_{Si} \times \Delta z$ (~ 17.5 µm) deeper steps in Si. In experiments, the onset of the modification for each group is measured to be separated by ~17.5 µm as expected. The fabrication parameters are, $r_0 = 7, E_{\mathrm{p}} = 7.3$ µJ, and $v = 1$ mm/s, the laser is raster scanned along the y axis. Sample is briefly etched before imaging. *Inset*: A zoomed SEM image representing the last line of a group, followed by 10 nano-planes of the next group. The observed variability of ~ 0.3 µm on the onset is ascribed to the repeatability of translation stage.

References

1. Boucher, P. *et al.* Generation of high conical angle Bessel-Gauss beams with reflective axicons. *Appl. Opt.* **57,** 6725–6728 (2018).
2. Lei, Y., Wang, H., Shayeganrad, G. & Kazansky, P. G. Ultrafast laser nanostructuring in transparent materials for beam shaping and data storage. *Opt. Mater. Express* **12,** 3327–3355 (2022).
3. Rudenko, A. *et al.* Spontaneous periodic ordering on the surface and in the bulk of dielectrics irradiated by ultrafast laser: a shared electromagnetic origin. *Sci. Rep.* **7,** 1–14 (2017).
4. Zhang, H. *et al.* Coherence in ultrafast laser-induced periodic surface structures. *Phys. Rev. B* **92,** 174109 (2015).
5. Oktem, B. *et al.* Nonlinear laser lithography for indefinitely large-area nanostructuring with femtosecond pulses. *Nat. Photon.* **7,** 897–901 (2013).
6. Yavuz, Ö. *et al.* Pattern guiding via 'structured noise'. *APS March Meeting* K47.005 Los Angeles USA (2018).
7. Yan, Z., Gao, J., Beresna, M. & Zhang, J. Near-field mediated 40 nm in-volume glass fabrication by femtosecond laser. *Adv. Opt. Materials* **10,** 2101676 (2022).
8. Kazansky, P. G. *et al.* High speed ultrafast laser anisotropic nanostructuring by energy deposition control via near-field enhancement. *Optica* **8,** 1365–1371 (2021).
9. Rudenko, A., Colombier, J.-P. & Itina, T. E. From random inhomogeneities to periodic nanostructures induced in bulk silica by ultrashort laser. *Phys. Rev. B* **93,** 075427 (2016).
10. Buschlinger, R., Nolte, S. & Peschel, U. Self-organized pattern formation in laser-induced multiphoton ionization. *Phys. Rev. B* **89,** 184306 (2014).
11. Li, Z.-Z. *et al.* O-FIB: far-field-induced near-field breakdown for direct nanowriting in an atmospheric environment. *Light. Sci. Appl.* **9,** 1–7 (2020).
12. Geng, J., Shi, L., Sun, X., Yan, W. & Qiu, M. Artificial seeds-regulated femtosecond laser plasmonic nanopatterning. *Laser Photonics Rev.* **16,** 2200232 (2022).
13. Chambonneau, M. *et al.* In-volume laser direct writing of silicon-challenges and opportunities. *Laser Photonics Rev.* **15,** 2100140 (2021).
14. Kononenko, V. V., Konov, V. V. & Dianov, E. M. Delocalization of femtosecond radiation in silicon. *Opt. Lett.* **37,** 3369–3371 (2012).
15. Grojo, D., Mouskeftaras, A., Delaporte, P. & Lei, S. Limitations to laser machining of silicon using femtosecond micro-Bessel beams in the infrared. *J. Appl. Phys.* **117,** 153105 (2015).
16. Tokel, O. *et al.* In-chip microstructures and photonic devices fabricated by nonlinear laser lithography deep inside silicon. *Nat. Photon.* **11,** 639–645 (2017).
17. Mermillod-Blondin, A. *et al.* Ultrafast laser nanostructuring in bulk silica, a slow microexplosion. *Optica* **4,** 951–958 (2017).
18. Courvoisier, F., Stoian, R. & Couairon, A. Ultrafast laser micro- and nano-processing with nondiffracting and curved beams. *Optics & Laser Technology* **80,** 125–137 (2016).
19. Török, P., Varga, P., Laczik, Z. & Booker, G. R. Electromagnetic diffraction of light focused through a planar interface between materials of mismatched refractive indices: an integral representation. *J. Opt. Soc. Am. A* **12,** 325–332 (1995).
20. Jesacher, A. & Booth, M. J. Parallel direct laser writing in three dimensions with spatially dependent aberration correction. *Opt. Express* **18,** 21090–21099 (2010).
21. Li, Q., Chambonneau, M., Chanal, M. & Grojo, D. Quantitative-phase microscopy of nanosecond laser-induced micro-modifications inside silicon. *Appl. Opt.* **55,** 9577–9583 (2016).
22. Saltik, A., Sabet, R. A. & Tokel, O. Refractive index and birefringence studies of laser-written structures buried inside silicon. *Laser Applications in Microelectronic and Optoelectronic Manufacturing (LAMOM) XXVIII* PC12408, PC124080E , SPIE, (2023).
23. Beresna, M., Bellouard, Y., Kazansky, P. & Champion, A. Stress distribution around femtosecond laser affected zones: effect of nanogratings orientation. *Opt. Express* **21,** 24942–24951 (2013).
24. Gdoutos, E. E. & Pericles, T. S. *Matrix Theory of Photoelasticity*. **11,** (Springer Series in Optical Sciences, 2013).

25. Stoehr, M., Gerlach, G., Härtling, T. & Schoenfelder, S. Analysis of photoelastic properties of monocrystalline silicon. *J. Sens. Sens. Syst.* **9,** 209–217 (2020).

26. Jacobsen, R. S. *et al.* Strained silicon as a new electro-optic material. *Nature* **441,** 199–202 (2006).

27. Bonati, C., Pintus, P. & Manganelli, C. L. Modeling of strain-induced Pockels effect in silicon. *Opt. Express* **23,** 28649–28666 (2015).

28. Munguía, J., Bremond, G., Bluet, J. M., Hartmann, J. M. & Mermoux, M. Strain dependence of indirect band gap for strained silicon on insulator wafers. *Appl. Phys. Lett.* **93,** 102101 (2008).

29. Turnali, A., Han, M. & Tokel, O. Laser-written depressed-cladding waveguides deep inside bulk silicon. *J. Opt. Soc. Am. B* **36,** 966–970 (2019).

30. Shafir, E. & Berkovic, G. Heterodyne interrogation scheme for Pi-phase-shifted fiber Bragg grating sensors. *Opt. Lett.* **44,** 514–517 (2019).

31. Thomas, T. R. Characterization of surface roughness. *Precision Engineering* **3,** 97–104 (1981).
